# Assessment of fatigue and recovery in elite cheerleaders prior to and during the ICU World Championships

**DOI:** 10.3389/fspor.2023.1105510

**Published:** 2023-03-06

**Authors:** Simon Gavanda, Christoph von Andrian-Werburg, Thimo Wiewelhove

**Affiliations:** ^1^ Department of Fitness & Health, IST University of Applied Sciences, Düsseldorf, Germany; ^2^ Institute for Cardiovascular Research and Sports Medicine, German Sport University Cologne, Cologne, Germany

**Keywords:** regeneration, cheerleading, stress, training load, monitoring

## Abstract

**Introduction:**

Little is known about the demands of competitive cheerleading. Therefore, the objective of this study was to assess fatigue and recovery during preparation for world championships.

**Methods:**

Fifteen participants from the German senior “All-Girl” and “Coed” national teams (nine males and six women) were recruited. Data were collected during the final preparation (T1 -T7) and competition days (C1 -C2). Heart rate variability (HRV) and resting heart rate (HR) were measured every morning. Data on training load, recovery, and stress (Short Scale for Recovery and Stress) were surveyed after training. Countermovement jump height (CMJ), sit-and-reach, and exercise-induced muscle damage (EMID) scores were taken in the afternoon.

**Results:**

There was a practically relevant decrease in CMJ (T2, T6). A trend for HR to increase (T5–C2) and HRV to decrease (T4, T6–C2) was evident. Through training, recovery decreased and recovered as C1 approached (mental performance: T2–T4 *p* = 0.004; T2–C1 *p* = 0.029; T3–T4 *p* = 0.029; emotional balance: T3–T4 *p* = 0.023; T3–C1 *p* = 0.014; general recovery status T1–T3 *p* = 0.008; T3–T4 *p* = 0.024; T3–C1 *p* = 0.041), whereas stress increased during the first days and returned to normal before C1 (emotional dysbalance: T2–T4 *p* = 0.014; T2–C1 *p* = 0.009; T3–T4 *p* = 0.023; T3–C1 *p* = 0.014). EMID scores increased for the upper and lower body between T3, T5–T7 (*p* ≤ 0.036) and T3, T6–T7 (*p* ≤ 0.047), respectively.

**Discussion:**

Pre-competition training led to substantial fatigue, and most markers indicate that athletes do not compete fully recovered. This could possibly be avoided by optimizing the training load or implementing recovery strategies.

## Introduction

Cheerleading has come a long way since Johnny Campbell led a crowd at a sporting event in cheers and chants for the first time at the end of the 19th century (1). In recent decades, cheerleading has evolved increasingly from a sideline activity into a competitive stand-alone sport (2,3) and has been regularly televised nationally in the USA, the sport's motherland, since 1982 (1). Today, the International Cheer Union (ICU), founded in 2004, comprises more than 116 nations, representing approximately 7.5 million athletes on all continents (4). The number of active cheerleaders is also increasing rapidly in Germany. According to the numbers of the German Olympic Sports Confederation, a growth of more than 20% was observed between 2018 and 2022 (5, 6). This is when many sport federations are experiencing a decline in membership due to the coronavirus pandemic.

Cheerleading as a competitive sport is done at the local, state, regional, national, and even international levels. One of these international competitions is the ICU World Championship, held annually in Orlando, Florida, where national teams from more than 20 countries compete. Teams must qualify on the first day of the championship for the final on the second day, when the world champion is determined. Competition routines of less than three minutes of duration, including gymnastic elements (“tumbling”), lifts (“stunts”), throws (“basket tosses”), and pyramids (7), are evaluated by judges based on several criteria, including difficulty and execution. This requires high levels of strength, power, flexibility, endurance, and balance for athletes to be successful (2, 8). In addition to athleticism, highly competitive cheerleading on an elite level requires precise coordination and timing between team members during the very dynamic competition routines.

Unfortunately, cheerleading is also a very dangerous sport, with catastrophic injuries (i.e., skull, brain, or spinal cord injuries) occurring (3, 9–11) in addition to more common injuries that also occur in other sports (i.e., strains, sprains, fractures, dislocations) (10, 12, 13). In addition to acute injuries, there is also a high prevalence of overuse injuries, which have been reported to account for 66% of all cheerleading-related injuries (14).

To prevent these injuries and to meet the high athletic demands of competitive cheerleading, it is recommended that athletes undertake additional strength training and conditioning (7, 10, 13, 15, 16). However, another often overlooked aspect of injury prevention in cheerleading could be stress and fatigue monitoring (17–19), since most cheerleading-related injuries occur during the months preparing for competition (10) and toward the end of a training session, typically lasting between 1.5 and 4 h (13, 20). Unfortunately, little is known about fatigue and stress during cheerleading competition preparation. However, data on the fatigue and stress of cheerleaders during the preparation for a competition could help to optimize training loads and to plan rest and recovery to prevent fatigue and injury (7).

For this reason, the goal of the study was to collect exploratory data on fatigue and recovery to understand more about the demands of competitive cheerleading on an elite level during the preparation phase for world championships. This may help with planning of training loads and implementing recovery strategies.

## Methods

The study participants were recruited from the German national cheerleading “All-Girl” [female athletes; ICU 2022 3rd place among 15 teams (ten teams in the highest category “All Girl - Premier” and five teams in the lower category “All Girl - Elite”).] and “Coed” [male and female athletes; ICU 2022 2nd place among 20 teams (eight teams in the highest category “Coed - Premier” and ten teams in the lower category “Coed - Elite”)] team (Cheerleading und Cheerperformance Verband Deutschland e.V.). Volunteers had to be healthy, over 18 years old, and currently rostered in the championship routine on the respective team of the ICU World Cheerleading Championships (Orlando, Florida). Substitute athletes were not considered participants, as their training load was significantly lower compared to starting athletes. Further exclusion criteria were cardiovascular diseases, especially cardiopulmonary diseases such as asthma or cardiac arrhythmias. Musculoskeletal injuries to the upper extremity, trunk, or legs within the last six months also led to exclusion from participation. All possible exclusion criteria were identified using a medical history form.

As part of the preparations for the world championships, multiple training camps took place in Germany. These were used to inform the athletes about the study duration and procedure, as well as the purpose and conduction of the planned measurements. Twenty-two athletes volunteered to participate in this study, of which 19 met the inclusion criteria. All participants gave their written informed consent to participate.

During the course of the study, two athletes dropped out due to orthopedic injuries suffered during cheer practice. Two other volunteers stopped the study for personal reasons. Therefore, 15 completed the study (“All-Girl”: two female flyers, two female bases; “Coed”: two female flyers, nine male bases). However, if the measurement time points of a parameter were missing, the data of the respective athlete were removed from further analysis. The descriptive data of the participants can be seen in Table 1.

**Table 1:** Descriptive data of the participants completing the study.

	*n*	Flyer	Bases	Age (y)	Height (cm)	Weight (kg)	BMI (kg/m^2^)
Total	15	4	12	25 ± 3	173 ± 12	73.7 ± 16.9	24.5 ± 3.2
Male	9	0	9	25 ± 3	180 ± 8	85.3 ± 10.1	26.2 ± 2.6
Female	6	3	3	25 ± 4	163 ± 9	60.1 ± 13.6	22.4 ± 2.6

The athletes arrived from Germany between April 8 and 13, 2022. Data collection was carried out during the final preparation for the competition (T1–T7) and the two competition days (C1–C2) of the ICU World Cheerleading Championships (April 14–22, 2022) in Orlando. Heart rate variability (HRV) and heart rate (HR) were measured every morning (T1–C2). Psychometric questionnaires were performed after training in the late afternoon (T1–C1). Performance testing (jump height), pain, and range of motion as indicators of fatigue delayed onset of muscle soreness were also done in the late afternoon (T1–T7).

An overview of the training days, times, and parts is shown in Table 2. After arrival in Orlando, cheerleading training took place on Days 1–3 (T1–T3) and 5–6 (T5–6). Day 4 (T4) was a day off. The training sessions of the “All-Girl” and “Coed” teams took place at the same time (10:00 am to 2:00 *p*.m.), but in different cheerleading gyms in the same location. In addition, on Day 7 (T7), a last one-hour training session took place in the competition arena. All athletes participated exclusively in their team training, as shown below, and did not participate in any other sporting activities.

**Table 2:** ICU World Cheerleading Championships.

		Day 1	Day 2	Day 3	Day 4	Day 5	Day 6	Day 7	Day 8	Day 9
		(T1)	(T2)	(T3)	(T4)	(T5)	(T6)	(T7)	(C1)	(C2)
Coed		Training	Training	Training	Rest	Training	Training	Open Practice	Competition	Competition
	Duration	150 min	270 min	240 min		270 min	180 min	60 min	60 min	60 min
	Part	Cheer	Warm up	Warm up		Warm Up	Warm Up	Cheer	Warm up	Warm up
			Cheer	9 x Run-through		Tumbling	6 x Run-through	2 x Run-through	Competition	Competition
			Pyramids	Baskets		8 x Run-through	3 x Full-outs	2 x Full-outs		
			Tumbling	Stunts		3 x Full-outs	Pyramids	1 x Run-throughs		
						Pyramids	Stunts	Pyramids		
All-Girl		Training	Training	Training	Rest	Training	Training	Open Practice	Competition	Competition
	Duration	150 min	210 min	240 min		210 min	225 min	60 min	60 min	60 min
	Part	Cheer	Warm up	Warm up		Warm up	Warm up	Cheer	Warm up	Warm up
			4 x Run-through	Tumbling		3 x Run-through	Tumbling	4 x Run-through	Competition	Competition
			Stunts	4 x Full-outs		4 x Full-outs	3 x Full-outs	Pyramids		
			Pyramids	Pyramids		Stunts	Pyramids			
			4 x Run-through	7 x Run-through		3 x Run-through	6 x Run-through			

All training sessions started with a 20–30 min cheerleading-specific warm-up routine. After the warm-up, either isolated elements of the competition routine (e.g., pyramids, tumbling, stunts, cheer, or basket tosses), a routine reduced to a few elements (“run-throughs”), or the complete routine was practiced (“full-out”). During the training sessions, there were several breaks to hydrate. Approximately in the middle of each training session, a longer break was given to provide snacks.

For the selection of valid monitoring parameters, the criteria “low time effort,” “uncomplicated,” and “cost-effective” had to be considered, since the focus of all athletes, the coaching staff, and the federation was successful participation at the world championships. Data collection was planned with the intention of not interfering with competition preparation and avoiding distracting athletes from their training as little as possible.

**Figure 1:**
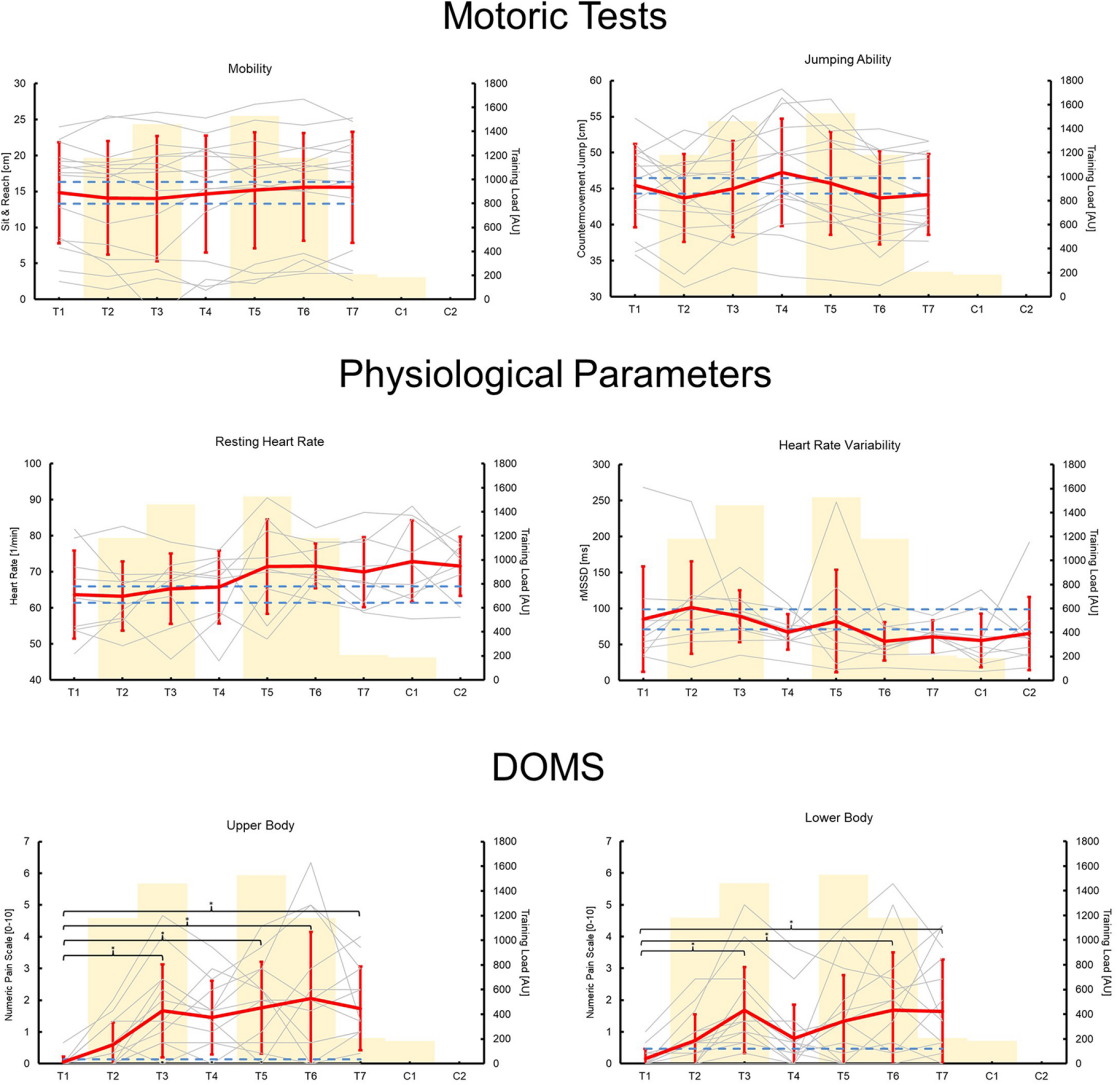
Mean (red lines) and individual (gray lines) changes in motor performance tests, physiological markers, and exercise-induced muscle damage (EIMD) score over time (training days T1–T7; competition days C1–C2). The yellow bars show a quantification of the internal training load (training load [arbitrary unit] = training duration [min] × session rate of perceived exertion [0–10]). The dashed blue lines indicate practically relevant changes of ≥0.2 effect sizes. Asterisks indicate statistically significant differences (*p* ≤ 0.05).

**Figure 2:**
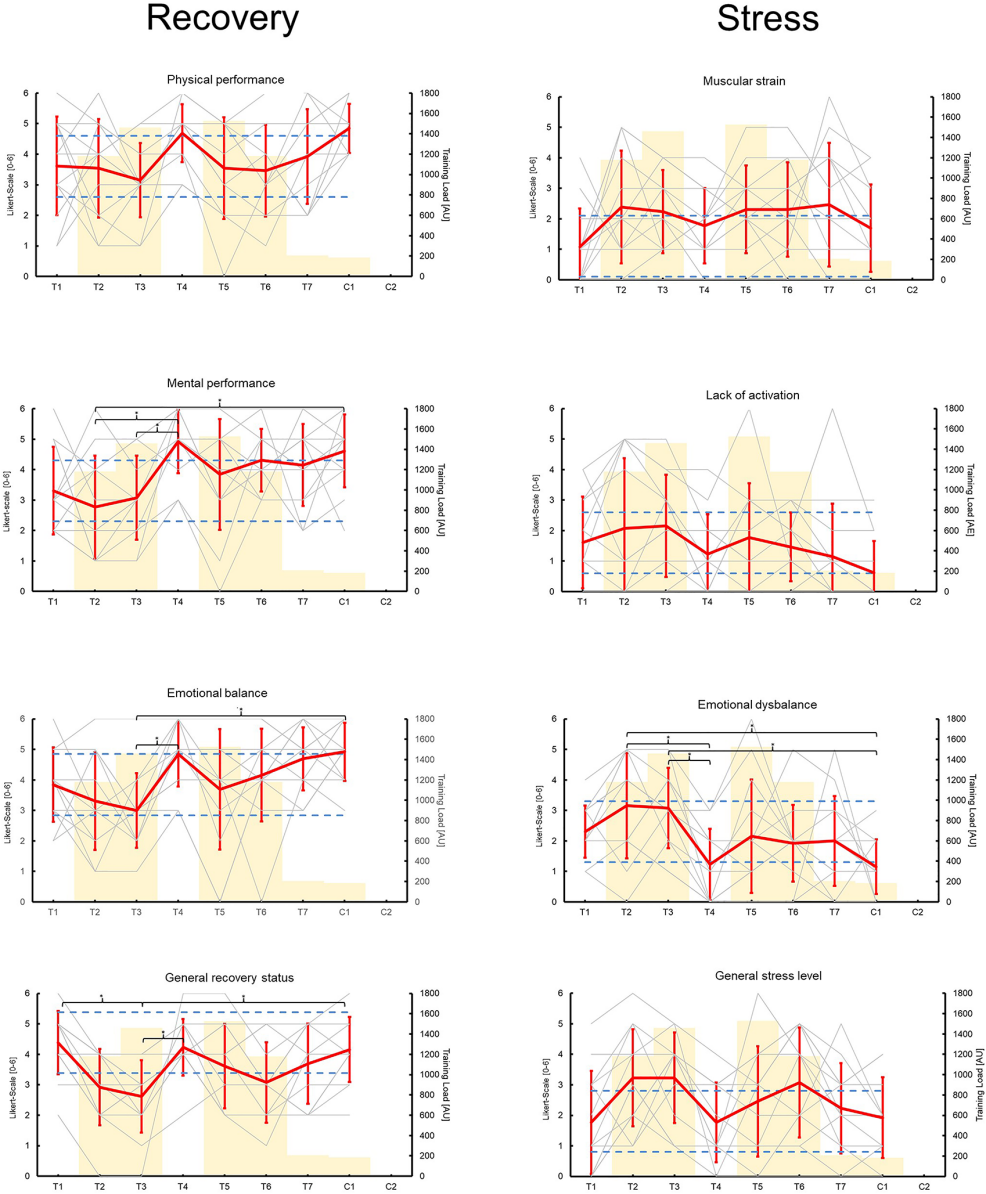
Mean (red lines) and individual (gray lines) changes of the short scale for recovery and stress (KEB) questionnaire items over time (training days T1–T7; competition days C1–C2). The yellow bars show a quantification of the internal training load (training load [arbitrary unit] = training duration [min] × session rate of perceived exertion [0–10]). The dashed blue lines indicate practically relevant changes of ±1 point on a Likert scale. Asterisks indicate statistically significant differences (*p* ≤ 0.05).

All measurements followed standardized procedures and were done identically at every time point (e.g., location, time of day, etc.). The data on the physiological markers were collected in the morning immediately after awakening. To measure the subjective rate of perceived exertion of a training session (sRPE), all athletes rated each training session using the CR-10 scale (21) immediately after practice during the one-hour drive back from the training location *via* the digital platform Survey Monkey questionnaire tool (Berlin, Germany) (22). This scale evaluates the sRPE from 0 (no exertion) to 10 (maximum exertion). The type and duration of training were documented by the research team (see Table 2). A quantification of the total training load was obtained by using the data on training intensity (CR-10) and volume (training duration) according to the method of Foster et al. (23). Using this method, the internal load of all study days was calculated (training load [TL] = training duration [min] × sRPE [0–10]) (24). Due to missing data, only 12 data sets could be evaluated for further analysis (see Figures 1, 2). After training, the assessment of recovery and stress was carried out using the German version of the Short Scale for Recovery and Stress (KEB). In the evening, the study participants came to the on-site testing station and performed motor performance tests and muscle pain assessments using an exercise-induced muscle damage (EIMD) score.

For the motor performance tests, the volunteers were divided into four small groups of five or six. A limited range of motion may be an indication of muscle destruction or edema, as seen in EIMD (25). Therefore, the sit-and-reach test was selected to determine the range of motion of the dorsally located leg and trunk muscles as an objective indicator of EIMD (26). A standardized sit-and-reach box (30 cm high) was used to obtain comparable and valid results. The athlete sat on the floor with extended knees and tried to bend the upper body forward as much as possible. The test measured how far the athlete could reach under the soles of the feet, with the fingertips and legs extended.

A counter movement jump (CMJ) is a practical monitoring tool, since a reduction in jump height is an indicator of neuromuscular fatigue (27). CMJ was measured using a wearable inertial measurement unit (CoRehab, Trentino, Italy), which has been shown to be accurate (28, 29). Three CMJs were performed with hands on their hips and 15 s of rest between jumps. The execution of the jumps was monitored by a member of the research team, and failed attempts (i.e., flexed knees during landing) were repeated. The mean value of the three jumps was used for further analysis. Data from 15 athletes were included in the final analysis.

Resting heart rate (HR) and variability of the time intervals between two heartbeats can provide an indication of cardiac autonomic function and the overall physiological state (30). The greater the variability of those time intervals, the more recovered or healthy a person is (31). In other words, increases in HR and reductions in daily HRV as a response to training may indicate reduced performance ability (30). All athletes were equipped with a chest strap measuring heart rate (Polar® H10, Polar, Kempele, Finland) and a mobile phone app (HRV4Training app, A.S.M.A. B.V., Amsterdam, Netherlands) on the day of arrival in Orlando. Participants received instructions on how to use the devices and perform the measurements correctly. For the measurements, the athletes had to measure both parameters for five minutes in a lying position directly after waking up, as previously described (32). HR and HRV data were stored in the HRV4Training app on the participants' phones. Raw data were collected after the last competition day. The root mean square of successive difference values (rMSSD) was used for further analysis. The rMSSD indicates the temporal differences between successive heartbeats. Due to technical problems or missing measurements, the HR and HRV datasets could only be analyzed from 9 of the 16 athletes.

To monitor recovery and stress states, the KEB (German: Kurzskala Erholung und Beanspruchung - KEB) questionnaire was developed. The original German version of the KEB is considered an economic, valid, and more important change-sensitive instrument for quantifing current recovery and stress (33). The KEB is based on a 7-point Likert scale (0: strongly disagree, 6: strongly agree) and consists of four items in each subscale of recovery (physical performance, mental performance, emotional balance, general recovery status) and stress (muscular strain, lack of activation, emotional dysbalance, general stress level) (33). At each time point, the participants completed a digital version of the KEB on their phones. Due to missing data, only data from 13 of the 16 athletes were included.

For subjectively measuring symptoms of EIMD, the sensation of muscle strain and soreness was used. For this, athletes were given a numeric pain scale ranging from 0 (normal, no pain) to 10 (worst imaginable muscle pain) (34) to rate perceived discomfort in three different conditions, each measured on the upper and lower extremities: (a) in a relaxed standing position; (b) under muscle contraction (athletes had to rate their soreness while performing three deep squats or three push-ups); (c) while a researcher palpated the muscle belly of the *quadriceps femoris* and *triceps brachii*. For further analysis, the mean pain value of the three conditions for the upper and lower extremities was calculated (EIMD score). Data from 14 to 15 athletes were used for further analysis of upper and lower extremity muscle soreness, respectively.

### Statistical analysis

Data in text and tables are expressed as mean (M) ± standard deviation (SD). The significance level of all statistical tests was set at *p* < 0.05. Statistical calculations were performed using Microsoft Excel 2016 (Microsoft Corporation, Redmond, WA, USA) and SPSS Statistics version 26.0 (IBM, Armonk, NY, USA). A one-way analysis of variances (ANOVA) was performed to determine changes in HRV, HR, KEB, pain, sit-and-reach distance, and jump height. Where necessary, Bonferroni *post hoc* analysis was performed. Effect sizes (ES) were calculated using Cohen's *d* = (M2–M1)/(SDpooled). For HRV, HR, EIMD scores, sit-and-reach distance, and jump height changes of >0.2 effect sizes (ES) were considered practically relevant. For KEB, changes of one point on the Likert scale compared to T1 were defined as practically relevant.

## Results

An overview of all the results and *p*-values for the time effects can be seen in Table 3. There were no significant time effects on sit-and-reach distance or jump height. However, there was a practically relevant decrease in CMJ in T2 and T6 and an increase in T4 compared to T1 (see Figure 1).

**Table 3:** ICU World Cheerleading Championships.

		T1	T2	T3	T4	T5	T6	T7	C1	C2	
Motor-performance testing	*n*	M ± SD	M ± SD	M ± SD	M ± SD	M ± SD	M ± SD	M ± SD			*p*
Sit & Reach [cm]	15	14.8 ± 7.0	14.1 ± 7.9	14 ± 8.7	14.6 ± 8.1	15.2 ± 8.1	15.6 ± 7.5	15.6 ± 7.7			0.996
CMJ [cm]	15	45.4 ± 5.8	43.7 ± 6.1	45 ± 6.7	47.3 ± 7.5	45.8 ± 7.2	43.7 ± 6.5	44.2 ± 5.6			0.739
Physiological markers	*n*	M ± SD	M ± SD	M ± SD	M ± SD	M ± SD	M ± SD	M ± SD	M ± SD	M ± SD	*p*
HR [1/min]	9	64 ± 12	63 ± 10	65 ± 10	66 ± 10	71 ± 13	72 ± 6	70 ± 10	73 ± 11	72 ± 8	0.277
HRV [rMSSD (ms)]	9	85 ± 73	101 ± 64	89 ± 36	67 ± 25	83 ± 71	54 ± 27	61 ± 23	55 ± 37	65 ± 51	0.422
KEB questionnaire	*n*	M ± SD	M ± SD	M ± SD	M ± SD	M ± SD	M ± SD	M ± SD	M ± SD		*p*
Physical performance	13	3.6 ± 1.6	3.5 ± 1.6	3.2 ± 1.2	4.7 ± 0.9	3.5 ± 1.7	3.5 ± 1.5	3.9 ± 1.6	4.8 ± 0.8		0.023
Mental performance	13	3.3 ± 1.4	2.8 ± 1.7	3.1 ± 1.4	4.9 ± 1.0	3.8 ± 1.8	4.3 ± 1.0	4.2 ± 1.3	4.6 ± 1.2		<0.001
Emotional balance	13	3.8 ± 1.2	3.3 ± 1.6	3.0 ± 1.2	4.8 ± 1.1	3.7 ± 2.0	4.2 ± 1.5	4.7 ± 1.0	4.9 ± 1.0		0.002
General recovery status	13	4.4 ± 1.0	2.9 ± 1.3	2.6 ± 1.2	4.2 ± 0.9	3.6 ± 1.4	3.1 ± 1.3	3.7 ± 1.3	4.2 ± 1.1		<0.001
Muscular strain	13	1.1 ± 1.3	2.4 ± 1.9	2.2 ± 1.4	1.8 ± 1.2	2.3 ± 1.4	2.3 ± 1.5	2.5 ± 2.0	1.7 ± 1.4		0.285
Lack of activation	13	1.6 ± 1.5	2.1 ± 2.3	2.2 ± 1.7	1.2 ± 1.3	1.8 ± 1.8	1.5 ± 1.1	1.2 ± 1.7	0.6 ± 1.0		0.250
Emotional dysbalance	13	2.3 ± 0.9	3.2 ± 1.7	3.1 ± 1.3	1.2 ± 1.2	2.2 ± 1.9	1.9 ± 1.3	2.0 ± 1.5	1.2 ± 0.9		0.001
General stress level	13	1.8 ± 1.7	3.2 ± 1.6	3.2 ± 1.5	1.8 ± 1.3	2.5 ± 1.8	3.1 ± 1.8	2.2 ± 1.5	1.9 ± 1.3		0.089
EIMD score	*n*	M ± SD	M ± SD	M ± SD	M ± SD	M ± SD	M ± SD	M ± SD			*p*
Upper body	14	0.05 ± 0.18	0.60 ± 0.71	1.67 ± 1.47	1.45 ± 1.16	1.76 ± 1.45	2.05 ± 2.10	1.74 ± 1.32			0.001
Lower body	15	0.16 ± 0.31	0.73 ± 0.85	1.69 ± 1.35	0.80 ± 1.05	1.33 ± 1.46	1.69 ± 1.82	1.64 ± 1.63			0.007

CMJ, countermovement jump height; HR, resting heart rate; HRV, heart rate variability; KEB, Short Scale for Recovery and Stress; EIMD, exercise-induced muscle damage; M, mean; SD, standard deviation.

One-way ANOVA showed no significant time effect on HR. However, there was a practically relevant trend for an increase in HR from T5 onward (ES: T1–T5 = 0.61; T1–T6 = 0.86; T1–T7 = 0.57; T1–C1 = 0.78; T1–C2 = 0.77) (see Figure 1). There was no significant time effect on HRV. However, a practically relevant trend for a decrease in HRV was found in T4 and T6 to C2 (ES: T1–T4 = −0.36; T1–T6 = −0.61; T1–T7 = −0.50; T1–C1 = −0.54; T1–C2 = −0.32) (see Figure 1).

The KEB showed significant time effects for physical performance, mental performance, emotional balance, general recovery status, and emotional dysbalance (*p*-values ≤ 0.023). Post-hoc analysis showed differences in mental performance (T2–T4 *p* = 0.004; T2–C1 *p* = 0.029; T3–T4 *p* = 0.029), emotional balance (T3–T4 *p* = 0.023; T3–C1 *p* = 0.014), general recovery status (T1–T3 *p* = 0.008; T3–T4 *p* = 0.024; T3–C1 *p* = 0.041), and emotional dysbalance (T2–T4 *p* = 0.014; T2–C1 *p* = 0.009; T3–T4 *p* = 0.023; T3–C1 *p* = 0.014). Furthermore, there were practically relevant changes over time for all items (±1 Likert point) (see Figure 2).

There were significant time effects for the EMID scores, with *p* ≤ 0.007. Post-hoc analysis showed an increase in EMID scores for the upper and lower body between T3, T5–T7 (*p* ≤ 0.036) and T3, T6–T7 (*p* ≤ 0.047), respectively, compared to T1.

## Discussion

The aim of this study was to collect exploratory data on fatigue and recovery to understand the demands of competitive cheerleading at an elite level during the days prior to and during the world championships. In summary, the results of the study show a practically relevant decrease in jumping performance, especially in the last two days before the championship. In addition, HR increases over time, and HRV decreases at the same time in a practically relevant way. This is indicative of fatigue effects at the central nervous level. Furthermore, most athletes enter competition with significantly increased muscle pain as an indicator of muscle damage, whereas most items of the KEB first deteriorate during the training phase, but then recover toward the competition days.

The fact that single markers show contradictory results is not surprising, since fatigue can affect different functional domains (for example, muscles and the peripheral, central nervous, and endocrine systems) (35). Therefore, it is recommended to use various markers of different domains for monitoring fatigue and recovery (e.g., subjective and objective markers, performance tests, physiological parameters) to get a better overall picture of the athlete's condition (35). Furthermore, there is a possibility that participants may have provided invalid information regarding their status of stress and recovery (social-desirability bias), as the more objective tests indicate greater fatigue than indicated in the questionnaires. Additional markers should be used in future studies with competitive cheerleaders to obtain more detailed information on fatigue and recovery in competitive cheerleading; examples include blood markers, such as creatine kinase, myoglobin, lactate, or saliva samples (e.g., cortisol, testosterone) (36–38).

Fatigue is a normal part of the training process, and, to a certain extent, it is the prerequisite for adaptations to take place. However, it is of utmost importance that athletes recover from the most important competition of the year to be able to perform at maximum capacity. The data presented show that the athletes competed in a somewhat fatigued state (central fatigue and muscular damage), although they did not perceive it that way. To avoid this in the future, several factors affecting fatigue can be discussed.

First, a higher overall fitness level would be advantageous for coping with high training loads. However, to the best of our knowledge, no recent study has investigated the physiological profiles of elite competitive cheerleaders. An old publication reported that cheerleaders are less fit than other athletes (39), whereas Thomas et al*.* (2) found a high fitness level in cheerleaders compared to other college sports. More recent studies are therefore necessary.

Second, reducing the total training load or spreading the load over a longer period of time, including tapering strategies, should have positive effects on athletes' recovery before competition. However, from a practical perspective, it is probably not possible to reduce the total training load (e.g., intensity) because in the days before the championship, the national teams need to practice the competition routine as a whole under very specific conditions. Otherwise, there is no time for this in the preparation period, as athletes live in different places in their home country and can train together only a few times a year. In addition, for logistical and financial constraints, it is not possible to modify the training schedule (e.g., training duration). Nevertheless, our data show the positive effects of implementing a rest day (T4) on athletes' recovery status (CMJ, KEB, lower body EIMD score) in the middle of the training week. Since the KEB item “muscular strain” and EIMD scores were significantly elevated before the championship it would probably be beneficial to implement additional rest days before competition, as EIMD and its associated negative effects on performance usually take several days to subside (25, 36).

If the training load cannot be reduced, a third possibility would be to optimize recovery between training sessions. Sleep has an essential physiological function and is certainly the most important factor in post-exercise recovery (40). In fact, it is well known that athletes commonly do not get enough sleep (quantity and quality) (41), and this may be exacerbated by factors such as travel and the associated jet lag (42). For this reason, future studies with competitive cheerleaders should evaluate sleep behavior and intervene when necessary. Adequate nutrition also plays an important role in recovery. The diet should be balanced, be dense in nutrients and energy, and meet the requirements of the sport (43). It is also possible to add supplements to the diet to help with recovery, such as proteins and essential amino acids (43). Unfortunately, there is currently a lack of data and recommendations regarding nutrition for competitive cheerleaders. Therefore, this should be the subject of future research.

Furthermore, recovery methods could be used to aid recovery to reduce the negative effects of a high training load. For example, cryotherapy (44) and foam rolling show positive effects on recovery (45), while active cool-down methods, such as stretching or low-intensity exercise, have little or no benefit (46). However, their application and effectiveness should be verified in a cheerleading-specific context. For example, despite potential recovery benefits, providing ice baths requires considerable logistical effort and therefore may not always be feasible. Ultimately, the effects of particular recovery methods should be considered individually, since not all athletes respond in the same way, and the potentially most effective measure loses its effect if athletes refuse to adopt it.

Like every study, the present study has its limitations. For example, only practical monitoring parameters were selected in order not to interfere with preparation for the world championship. Although these are considered valid and practically relevant (35), future studies could complement them with more sophisticated measures, such as blood and hormonal markers, as mentioned above. In addition, the presented HRV data must be viewed with caution, since no individual baseline resting HRV of the athletes was established prior to this study. However, it is recommended to track HRV over a longer period (“longitudinal tracking”) to better understand how individual athletes respond to training stresses (30, 47). The reason for this is that HRV depends not only on training variables (e.g., exercise duration and intensity) but also on many other factors, such as training status, age, sex, emotional and mental stressors, or sleep (30). Therefore, future studies should collect HRV data over at least three days prior to the actual investigation to identify changes in HRV due to exercise stress more accurately (30). Nevertheless, even with longer surveys, it is not possible to differentiate between fatigue from exercise or other factors by HRV measurement. For this reason, conclusions should not be drawn based solely on HRV data and additional parameters should be surveyed. However, if most of these parameters indicate a trend towards fatigue, as in the present work, maladaptive stress is likely. On the other hand, the strength of the present study is that it is the first of its kind in the field of competitive cheerleading and cheerleaders from the best teams in the world in their respective categories were recruited as participants (“Coed” 2nd place; “All-girl” 3rd place). In general, there is a high need for research on the physiological requirements of this unique sport. Understanding the demands of competitive cheerleading can help to develop sport-specific strength and conditioning training programs and allow for better planning of training loads in order to prevent falls and injuries (10, 15, 16), since it is known that most cheerleading-related injuries occur during preparing for competition and near the end of a training session (10, 20). Furthermore, this study also may help with choosing and timing of appropriate recovery strategies during competition preparation. However, this topic should be usefully explored in further research.

In summary, this observational study shows that the typical training of cheerleading national teams prior to the world championships leads to substantial fatigue, and that most markers indicate that athletes do not go into the competition fully recovered. Therefore, it is necessary to modify the training load during preparation for the world championships and/or to implement adequate recovery measures to achieve maximum performance in competition. Whether the latter can be implemented effectively in the practice of competition cheerleading should be investigated in future work.

## Data Availability

The raw data supporting the conclusions of this article will be made available by the authors, without undue reservation.

## References

[1] International Cheer Union. “History of Cheer”. Available at: https://cheerunion.org.ismmedia.com/ISM3/std-content/repos/Top/2013_Website/About%20Us/Documents/ICU_History-Of-Cheer.pdf (Accessed: October 27, 2022) (2013).

[2] Thomas DQ Seegmiller JG Cook TL Young BA. Physiologic profile of the fitness status of collegiate cheerleaders. J Strength Cond Res. (2004) 18(2):252–4. 10.1519/R-12802.1 15142007

[3] LaBella CR Mjaanes J. Cheerleading injuries: epidemiology and recommendations for prevention. Pediatrics. (2012) 130(5):966–71. 10.1542/peds.2012-2480 23090348

[4] International Cheer Union. “What is the ICU”. Available at: https://cheerunion.org/about/about/ (Accessed: October 27, 2022) (2022).

[5] German Olympic Sports Confederation. “Bestandserhebung 2018: Fassung vom 1. November 2018”. Available at: https://cdn.dosb.de/user_upload/www.dosb.de/uber_uns/Bestandserhebung/BE-Heft_2018.pdf (Accessed: October 28, 2022) (2018).

[6] German Olympic Sports Confederation. “Bestandserhebung 2022: Fassung vom 1.10.2022”. Available at: https://cdn.dosb.de/user_upload/www.dosb.de/uber_uns/Bestandserhebung/BE-Heft_2022.pdf (Accessed: October 28, 2022) (2022).

[7] Xu AL Beck JJ Sweeney EA Severson MN Page AS Lee RJ. Understanding the cheerleader as an orthopaedic patient: an evidence-based review of the literature. Orthop J Sports Med. (2022) 10(1):23259671211067222. 10.1177/23259671211067222 35083360 PMC8785319

[8] Currie DW Fields SK Patterson MJ Comstock RD. Cheerleading injuries in United States high schools. Pediatrics. (2016) 137(1):1–9. 10.1542/peds.2015-2447 26729538

[9] Boden BP Tacchetti R Mueller FO. Catastrophic cheerleading injuries. Am J Sports Med. (2003) 31(6):881–8. 10.1177/03635465030310062501 14623653

[10] Bagnulo A. Cheerleading injuries: a narrative review of the literature. J Can Chiropr Assoc. (2012) 56(4):292–8. 23204573 PMC3501916

[11] Xu AL Suresh KV Lee RJ. Progress in cheerleading safety: update on the epidemiology of cheerleading injuries presenting to US emergency departments, 2010-2019. Orthop J Sports Med. (2021) 9(10):1–8. 10.1177/23259671211038895 PMC8524718 34676270

[12] Shields BJ Smith GA. Cheerleading-related injuries in the United States: a prospective surveillance study. J Athl Train. (2009a) 44(6):567–77. 10.4085/1062-6050-44.6.567 19911082 PMC2775357

[13] Shields BJ Smith GA. Epidemiology of cheerleading fall-related injuries in the United States. J Athl Train. (2009b) 44(6):578–85. 10.4085/1062-6050-44.6.578 19911083 PMC2775358

[14] Stracciolini A Casciano R Friedman HL Meehan WP Micheli LJ. A closer look at overuse injuries in the pediatric athlete. Clin J Sport Med. (2015) 25(1):30–5. 10.1097/JSM.0000000000000105 24926911

[15] Mueller FO. Cheerleading injuries and safety. J Athl Train. (2009) 44(6):565–6. 10.4085/1062-6050-44.6.565 19911081 PMC2775356

[16] Waters N. What goes up must come down! A primary care approach to preventing injuries amongst highflying cheerleaders. J Am Assoc Nurse Pract. (2013) 25(2):55–64. 10.1111/1745-7599.12000 23347241

[17] Kellmann M. Preventing overtraining in athletes in high-intensity sports and stress/recovery monitoring. Scand J Med Sci Sports. (2010) 20(2):95–102. 10.1111/j.1600-0838.2010.01192.x 20840567

[18] Schwellnus M Soligard T Alonso J-M Bahr R Clarsen B Dijkstra HP How much is too much? (part 2) international olympic committee consensus statement on load in sport and risk of illness. Br J Sports Med. (2016) 50(17):1,043–52. 10.1136/bjsports-2016-096572 27535991 PMC5013087

[19] Soligard T Schwellnus M Alonso J-M Bahr R Clarsen B Dijkstra HP How much is too much? (part 1) international Olympic committee consensus statement on load in sport and risk of injury. Br J Sports Med. (2016) 50(17):1,030–41. 10.1136/bjsports-2016-096581 26702012

[20] Jacobson BH Redus B Palmer T. An assessment of injuries in college cheerleading: distribution, frequency, and associated factors. Br J Sports Med. (2005) 39(4):237–40. 10.1136/bjsm.2004.014605 15793095 PMC1725182

[21] Borg GA. Psychophysical bases of perceived exertion. Med Sci Sports Exercise. (1982) 14(5):377–81. 10.1249/00005768-198205000-00012 7154893

[22] Brink MS Visscher C Arends S Zwerver J Post WJ Lemmink KA. Monitoring stress and recovery: new insights for the prevention of injuries and illnesses in elite youth soccer players. Br J Sports Med. (2010) 44(11):809–15. 10.1136/bjsm.2009.069476 20511621

[23] Foster C Florhaug JA Franklin J Gottschall L Hrovatin LA Parker S A new approach to monitoring exercise training. J Strength Cond Res. (2001) 15(1):109–15. 10.1519/00124278-200102000-00019 11708692

[24] Halson SL. Monitoring training load to understand fatigue in athletes. Sports Med. (2014) 44(2):139–47. 10.1007/s40279-014-0253-z PMC4213373 25200666

[25] Cheung K Hume P Maxwell L. Delayed onset muscle soreness: treatment strategies and performance factors. Sports Med. (2003) 33(2):145–64. 10.2165/00007256-200333020-00005 12617692

[26] Mayorga-Vega D Merino-Marban R Viciana J. Criterion-related validity of sit-and-reach tests for estimating hamstring and lumbar extensibility: a meta-analysis. J Sports Sci Med. (2014) 13(1):1–14. 24570599 PMC3918544

[27] Gathercole R Sporer B Stellingwerff T Sleivert G. Alternative countermovement-jump analysis to quantify acute neuromuscular fatigue. Int J Sports Physiol Perform. (2015) 10(1):84–92. 10.1123/ijspp.2013-0413 24912201

[28] Milosevic B Farella E. Wearable inertial sensor for jump performance analysis. Proceedings of the 2015 workshop on wearable systems and applications; May 18, 2015; Florence, Italy. New York, NY, USA: ACM (2015). p. 15–20

[29] Nielsen ET Jørgensen PB Mechlenburg I Sørensen H. Validation of an inertial measurement unit to determine countermovement jump height. Asia-Pacific J Sports Med Arthrosc Rehabil Technol. (2019) 16:8–13. 10.1016/j.asmart.2018.09.002 PMC6445523 30984557

[30] Lundstrom CJ Foreman NA Biltz G. Practices and applications of heart rate variability monitoring in endurance athletes. Int J Sports Med. (2023) 44(01):9–19. 10.1055/a-1864-9726 35853460

[31] Joyce D Barrett M. State of the science: heart rate variability in health and disease. BMJ Support Palliat Care. (2019) 9(3):274–6. 10.1136/bmjspcare-2018-001588 30446491

[32] Kiviniemi AM Hautala AJ Kinnunen H Nissilä J Virtanen P Karjalainen J Daily exercise prescription on the basis of HR variability among men and women. Med Sci Sports Exercise. (2010) 42(7):1,355–63. 10.1249/MSS.0b013e3181cd5f39 20575165

[33] Hitzschke B Kölling S Ferrauti A Meyer T Pfeiffer M Kellmann M. Entwicklung der kurzskala zur erfassung von erholung und beanspruchung im sport (KEB). Z Sport psychol. (2015) 22(4):146–62. 10.1026/1612-5010/a000150

[34] Breivik EK Björnsson GA Skovlund E. A comparison of pain rating scales by sampling from clinical trial data. Clin J Pain. (2000) 16(1):22–8. 10.1097/00002508-200003000-00005 10741815

[35] Meyer T Ferrauti A Kellmann M Pfeiffer M. Regenerationsmanagement im spitzensport: REGman – ergebnisse und handlungsempfehlungen. Köln: Sportverlag Strauß (2016).

[36] Clarkson PM Hubal MJ. Exercise-induced muscle damage in humans. Am J Phys Med Rehabil. (2002) 81(11):52–69. 10.1097/00002060-200211001-00007 12409811

[37] Brancaccio P Lippi G Maffulli N. Biochemical markers of muscular damage. Clin Chem Lab Med. (2010) 48(6):757–67. 10.1515/CCLM.2010.179 20518645

[38] Kraemer WJ Ratamess NA Nindl BC. Recovery responses of testosterone, growth hormone, and IGF-1 after resistance exercise. J Appl Physiol. (2017) 122(3):549–58. 10.1152/japplphysiol.00599.2016 27856715

[39] Cieslak T Engels HJ Nelson J Kolokouri I Wirth JC. Body composition and isokinetic knee strength of female high school varsity cheerleaders. Med Sci Sports Exerc. (2001) 33(5):247. 10.1097/00005768-200105001-01390

[40] Vitale KC Owens R Hopkins SR Malhotra A. Sleep hygiene for optimizing recovery in athletes: review and recommendations. Int J Sports Med. (2019) 40(8):535–43. 10.1055/a-0905-3103 31288293 PMC6988893

[41] Simpson NS Gibbs EL Matheson GO. Optimizing sleep to maximize performance: implications and recommendations for elite athletes. Scand J Med Sci Sports. (2017) 27(3):266–74. 10.1111/sms.12703 27367265

[42] van Janse Rensburg DC van Jansen Rensburg A Fowler PM Bender AM Stevens D Sullivan KO Managing travel fatigue and jet lag in athletes: a review and consensus statement. Sports Medicine. (2021) 51(10):2029–50. 10.1007/s40279-021-01502-0 34263388 PMC8279034

[43] Kerksick CM Wilborn CD Roberts MD Smith-Ryan A Kleiner SM Jäger R ISSN Exercise & sports nutrition review update: research & recommendations. J Int Soc Sports Nutr. (2018) 15(1):38. 10.1186/s12970-018-0242-y 30068354 PMC6090881

[44] Kwiecien SY McHugh MP. The cold truth: the role of cryotherapy in the treatment of injury and recovery from exercise. Eur J Appl Physiol. (2021) 121(8):2125–42. 10.1007/s00421-021-04683-8 33877402

[45] Wiewelhove T Döweling A Schneider C Hottenrott L Meyer T Kellmann M A meta-analysis of the effects of foam rolling on performance and recovery. Front Physiol. (2019) 376:1–15. 10.3389/fphys.2019.00376 PMC6465761 31024339

[46] van Hooren B Peake JM. Do we need a cool-down after exercise? A narrative review of the psychophysiological effects and the effects on performance, injuries and the long-term adaptive response. Sports Medicine. (2018) 48(7):1575–95. 10.1007/s40279-018-0916-2 29663142 PMC5999142

[47] Plews DJ Laursen PB Stanley J Kilding AE Buchheit M. Training adaptation and heart rate variability in elite endurance athletes: opening the door to effective monitoring. Sports Med. (2013) 43:773–81. 10.1007/s40279-013-0071-8 23852425

